# Decreased axial diffusivity in the superior longitudinal fasciculus correlates with fronto-parietal functional connectivity in psychotic patients with persistent delusions

**DOI:** 10.1192/j.eurpsy.2023.1933

**Published:** 2023-07-19

**Authors:** A. S. Tomyshev, Y. Panikratova, E. Abdullina, I. Lebedeva, P. Iuzbashian, K. Dmitrenko, G. Kostyuk, A. Andriushchenko, D. Romanov, A. Smulevich

**Affiliations:** 1 Mental Health Research Center; 2 I.M. Sechenov First Moscow State Medical University; 3 Mental-health Clinic No.1 named after N.A. Alexeev, Moscow, Russian Federation

## Abstract

**Introduction:**

There is growing evidence to suggest that delusions in schizophrenia-spectrum disorders are associated with altered brain connectivity. Disruptions in long association fibers, such as the superior longitudinal fasciculus, are among the most consistent findings in psychosis. However, functional connectivity (FC) correlates of such structural alterations and their implications in delusional symptoms remains unclear.

**Objectives:**

The study used a hypothesis-driven approach and aimed at exploring structural connectivity (SC) disruptions of the left superior longitudinal fasciculus (part with parietal terminations, SLFP) and their FC correlates in a group of psychotic patients with persistent delusions across diagnostic categories within the schizophrenia-spectrum.

**Methods:**

Sixteen right-handed patients (23.1-53.8 years, mean age 39.6±8.5, 44% females) with delusional disorder (DD, n=10) and schizophrenia (SCZ, n=6), presenting with persistent delusions, and 16 matched healthy controls (23.0-56.4 years, mean age 38.9±11.1, 44%females) underwent diffusion-weighted 3T MRI (DW-MRI), while patients additionally underwent resting-state 3T fMRI (rsfMRI). DW-MRI data were processed via FreeSurfer6.0 and TRACULA to derive axial (AD), radial (RD) diffusivities and fractional anisotropy (FA) for left SLFP. rsfMRI data were processed with SPM12 and Conn v19c to calculate ROI-to-ROI FC between lateral prefrontal and inferior parietal components of the frontoparietal network (FPCN) according to Yeo atlas (Yeo *et al*. J Neurophysiol. 2011; 106(3) 1125-65), which is sought to represent cortical projections of the SLFP (Image). Partial rank-based correlation analysis (with age and sex as covariates, ppcor v1.1, R v4.2.1) was used to explore the associations between SC and FC measures involving the SLFP, PANSS and BABS scores.

**Results:**

Compared to healthy controls, patients showed decreased AD in left SLFP [F(1, 28)=14.9, p=0.0006; Cohen’s *d* = −1.3, 95% CI: −2.1 to −0.5]. No RD or FA alterations were found. We revealed a correlation between AD in left SLFP and fronto-parietal FC within the FPN (r = 0.58, p = 0.031) in patients. Correlation between FC and PANSS total score (r = −0.54, p = 0.045) did not survived correction for multiple comparisons. No other correlations between SC or FC, chlorpromazine equivalents and clinical scores were revealed.

**Image:**

**Figure:**
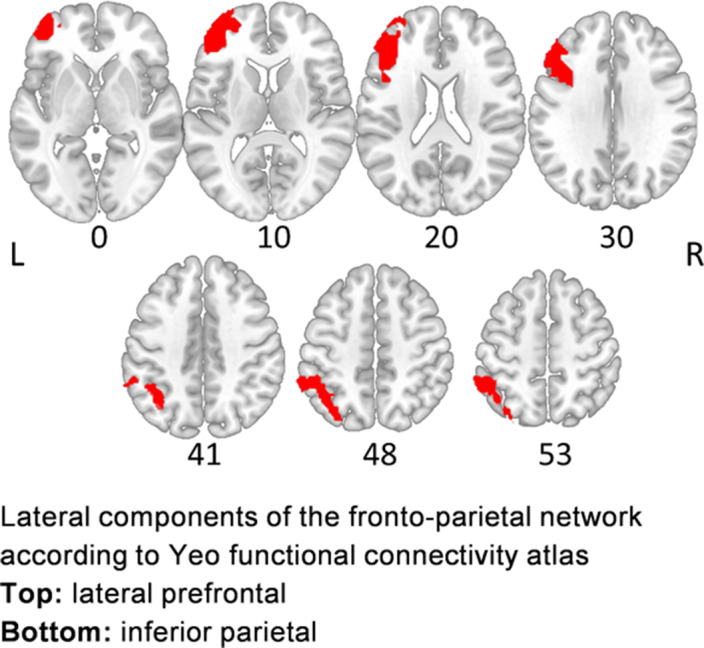

**Conclusions:**

The findings suggest that the structural connectivity disruptions of the SLFP may mediate FC strength within the FPN in patients with persistent delusions. However the limited sample size and the lack of correlations between connectivity measures and clinical scores do not allow to conclude definitely whether the revealed structural-functional connectivity pattern underlies delusional symptoms, which should be elucidated via further research.

*This study was supported by RFBR grant 21-515-12007*

**Disclosure of Interest:**

None Declared

